# Heart rate variability-based prediction of early cardiotoxicity in breast-cancer patients treated with anthracyclines and trastuzumab

**DOI:** 10.1186/s40959-024-00236-y

**Published:** 2024-05-29

**Authors:** Santiago Luna-Alcala, Adrián Espejel-Guzmán, Claudia Lerma, Paula Leon, Enrique C. Guerra, Jose Rodrigo Espinosa Fernández, Pavel Martinez-Dominguez, Javier Serrano-Roman, Aldo Cabello-Ganem, Alexis D. Aparicio-Ortiz, Candace Keirns, Abel Lerma, Maria Jose Santa Ana-Bayona, Nilda Espinola-Zavaleta

**Affiliations:** 1 Department of Nuclear Cardiology, National Institute of Cardiology Ignacio Chavez, Colonia Seccion XVI, Juan Badiano No 1, Colonia Seccion XVI, Tlalpan, Mexico City, 14080 Mexico; 2 Mexican Social Security Institute IMSS, Mexico City, Mexico; 3 Department of Molecular Biology, National Institute of Cardiology Ignacio Chavez, Colonia Seccion XVI, Juan Badiano 1, Tlalpan, Mexico City, 14080 Mexico; 4 Department of Electrical Engineering, Universidad Autónoma Metropolitana, Unidad Iztapalapa, Mexico City, 09340 México; 5 Breast Cancer Department, National Institute of Cancer, Mexico City, Mexico; 6 Shelby County Health Department, Memphis, TN USA; 7 Institute of Health Sciences, Universidad Autónoma del Estado de Hidalgo, San Agustín Tlaxiaca, 42160 Mexico; 8 Department of Echocardiography, ABC Medical Center, I.A.P, Mexico City, Mexico

**Keywords:** Heart rate variability, Breast cancer, Anthracyclines, Trastuzumab, Sympathetic, Parasympathetic

## Abstract

**Background:**

Cardiotoxicity is a recognized complication in breast cancer (BC) patients undergoing chemotherapy with anthracyclines with or without trastuzumab. However, the prognostic value of heart rate variability (HRV) indexes for early cardiotoxicity development remains unknown.

**Methods:**

Fifty BC patients underwent TTE assessment before and three months after chemotherapy. HRV indexes were obtained from continuous electrocardiograms in supine position with spontaneous breathing, active standing, and supine position with controlled breathing. The magnitude of change (Δ) between supine-standing and supine-controlled breathing was calculated. Variables were compared using t-test or ANOVA. Cardiotoxicity predictive value was assessed by ROC curve analysis. A *p* value of < 0.05 was considered significant.

**Results:**

TTE revealed reduced left atrial conduit strain in the cardiotoxicity group. Mean heart rate increased during all maneuvers at follow-up, with no differences in HRV indexes between patients with or without cardiotoxicity. However, a lower Δ in supine-controlled breathing of several HRV indexes predicted early cardiotoxicity identified by echocardiography (e.g. SDNN ≤ -8.44 ms: Sensitivity = 75%, Specificity = 69%).

**Conclusions:**

BC patients treated with chemotherapy maintain cardiac autonomic responses to physiological stimuli after 3 months of chemotherapy. However, a lower Δ during active standing and controlled breathing before chemotherapy may predict early cardiotoxicity.

**Supplementary Information:**

The online version contains supplementary material available at 10.1186/s40959-024-00236-y.

## Introduction

Breast cancer (BC) is the most common diagnosed cancer worldwide with 2.3 million new cases in 2020 (11.7% of all new cancer cases) and 685,000 deaths [1]. By 2040, an annual reduction of 2.5% in global BC mortality is expected [2].

Chemotherapy enhances survival rates but carries cardiotoxicity risk, a clinically silent complication that can affect up to 27% of patients receiving trastuzumab chemotherapy and as high as 34% when combined with anthracyclines [3, 4]. Cumulative dosage and the administration method of anthracyclines cause irreversible cardiotoxicity while trastuzumab’s cardiotoxicity is reversible [5].

After chemotherapy, early cardiac injury may manifest as autonomic dysfunction, preceding ventricular dysfunction and reduced left ventricular ejection fraction (LVEF) [6]. Close monitoring is essential for the detection of potential adverse consequences, including cardiac dysfunction [7].

Current definitions of cancer therapy-related cardiovascular dysfunction (CTRCD) include cardiac injury, cardiomyopathy, and heart failure. Diagnostic variables include diminished LVEF and global longitudinal strain (GLS) and/or new increases in cardiac biomarkers. Anthracyclines or *HER2*-targeted therapy patients need regular monitoring to detect CTRCD based on clinical findings, biomarkers, and transthoracic echocardiography (TTE) [8].

Lakoski et al. (2015) reported therapy-induced injuries causing adverse cardiovascular and lifestyle impacts that led to increased sympathetic nervous system (SNS) activation and decreased parasympathetic nervous system (PSNS) activation [6]. This autonomic dysfunction can result in cardiac manifestations as hypertension, myocardial ischemia, heart failure, cardiac arrhythmias, and sudden cardiac death [9].

Initial investigations show that pharmacological interventions (β-blockers) that target increased heart rate variability (HRV) and normalize autonomic function can mitigate chemotherapy’s negative effects on cardiac function [10].

HRV indexes and their correlation with TTE parameters have not been examined in BC patients on anthracyclines or *HER2*-targeted therapy. In a two-year prospective observational study of *HER2 +* BC patients treated with trastuzumab, Feng et al. analyzed the association of HRV changes and cardiotoxicity. They found that deceleration capacity (DC) had higher sensitivity and specificity in predicting cardiotoxicity than baseline LVEF, and reduced DC correlated with earlier onset [11]. However, another prospective study with BC patients treated with epirubicin showed no persistent alterations in diastolic function and HRV [12]. There are no studies assessing the cardiac autonomic response by controlling physiological stimuli (such as active standing and rhythmic breathing) in BC patients.

In this study, we evaluate cardiac autonomic responses to physiological stimuli in BC patients treated with anthracyclines with or without trastuzumab and assess the predictive value of both HRV indexes and TTE parameters for early cardiotoxicity.

## Methods

### Study design

**Fig. 1:**
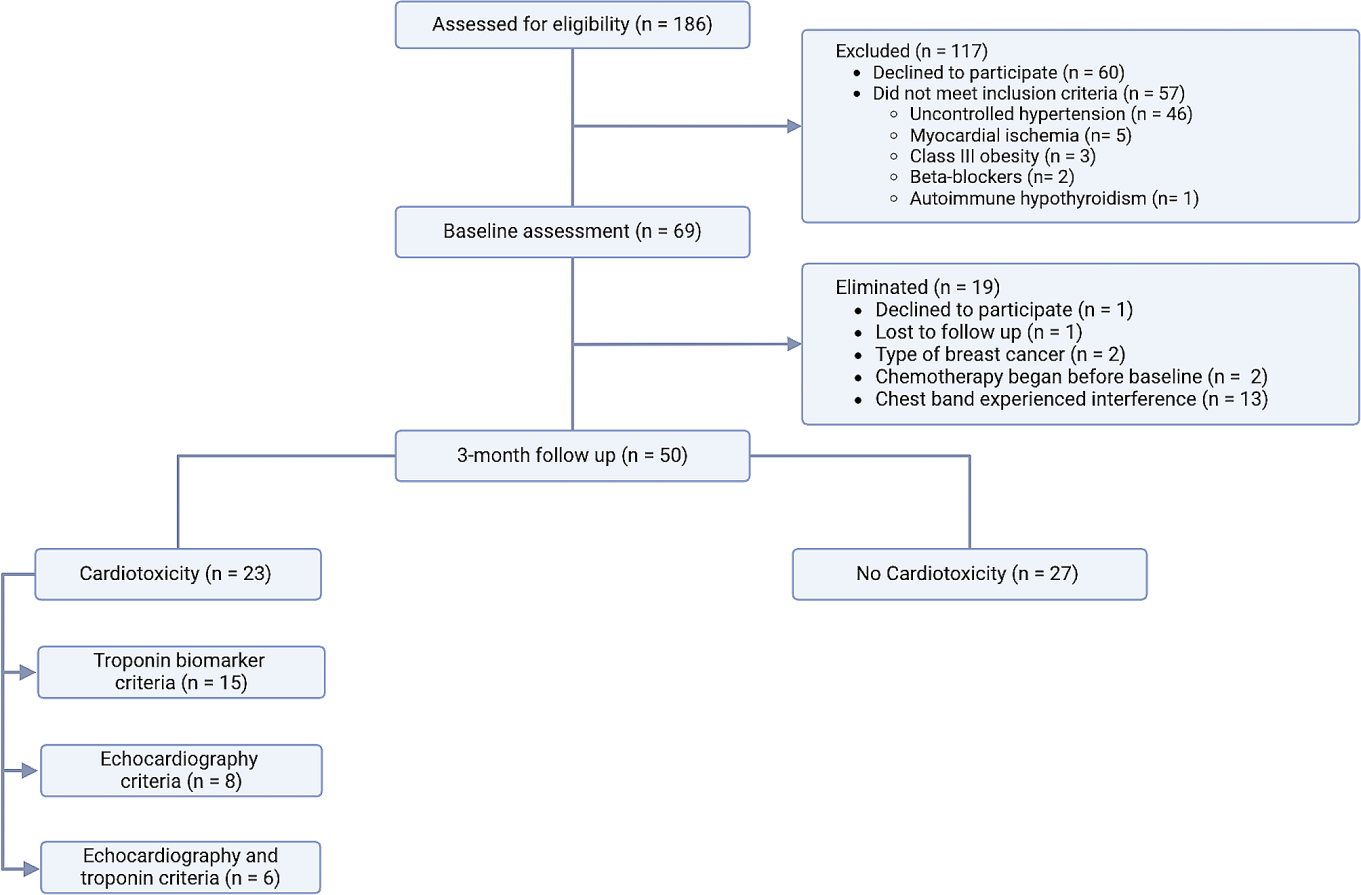
Overview of patient screening and enrollment. Caption: This figure illustrates the participant selection process from the breast cancer patients’ cohort, including its exclusion criteria for baseline assessment and elimination criteria for the three-month follow-up. The number of participants who met the cardiotoxicity criteria, either with troponins or echocardiography criteria or both, are indicated on the left side

From February 2022 to May 2023, a prospective cohort study was conducted in our institution with BC patients referred from a cancer referral center. Inclusion criteria comprised female BC patients eligible for chemotherapy with anthracyclines, trastuzumab, or both. Exclusion criteria included a body mass index (BMI) > 40 Kg/m^2^, uncontrolled systemic hypertension or current treatment with β-blockers, myocardial ischemia, chronic renal disease, and autoimmune disease. Figure 1 depicts the selection process flow chart. Initially, baseline assessments were conducted for sixty-nine patients before chemotherapy. Subsequently, during the follow-up period, nineteen patients were eliminated. After three-months of chemotherapy, fifty patients underwent assessments. The study protocol was approved by the Research and Ethics Committee of our Institution (protocol number 21-1259). Written informed consent for study enrollment was obtained from all patients.

### Experimental design

Enrolled patients underwent a baseline assessment, including TTE and HRV studies, along with a medical history focused on significant comorbidities, vital signs, and high-sensitivity troponin T (hsTnT) measurement. After three-months, they were reassessed with TTE and HRV studies, and medical histories, vital signs and hsTnT measurements were conducted.

### Chemotherapy regimen

Forty-eight patients received 4 cycles of AC (doxorubicin [Adriamycin] + cyclophosphamide [Cytoxan]) chemotherapy, with an anthracycline cumulative dose of 240 mg. Only two patients received 1 cycle of AC with an anthracycline cumulative dose of 60 mg. Each chemotherapy cycle lasted one day, with an intravenous (IV) drip of 60 mg/m^2^ doxorubicin and 600 mg/m^2^ cyclophosphamide. Each cycle was repeated every 3 weeks for a total of 4 cycles. Twenty-one *HER2* + patients subsequently received Paclitaxel (Taxol) and concurrent Trastuzumab (Herceptin). Each of these cycles involved an IV drip of 80 mg/m^2^ Paclitaxel once weekly for 12 weeks. This was accompanied by an IV loading dose of 4 mg/kg Trastuzumab, followed by 2 mg/kg IV once weekly or 6 mg/kg IV once every 3 weeks, completing 1 year of treatment [13].

### Electrocardiogram recording and HRV indices

A continuous electrocardiogram (ECG) was recorded using a chest band (Bioharness 3.0, Zephyr Technology, Annapolis, MD, USA). The QRS complex for each heartbeat was identified using our validated custom-made computer program [14]. Participants initiated the recording protocol by lying down, silent and with closed eyes.

**Fig. 2:**
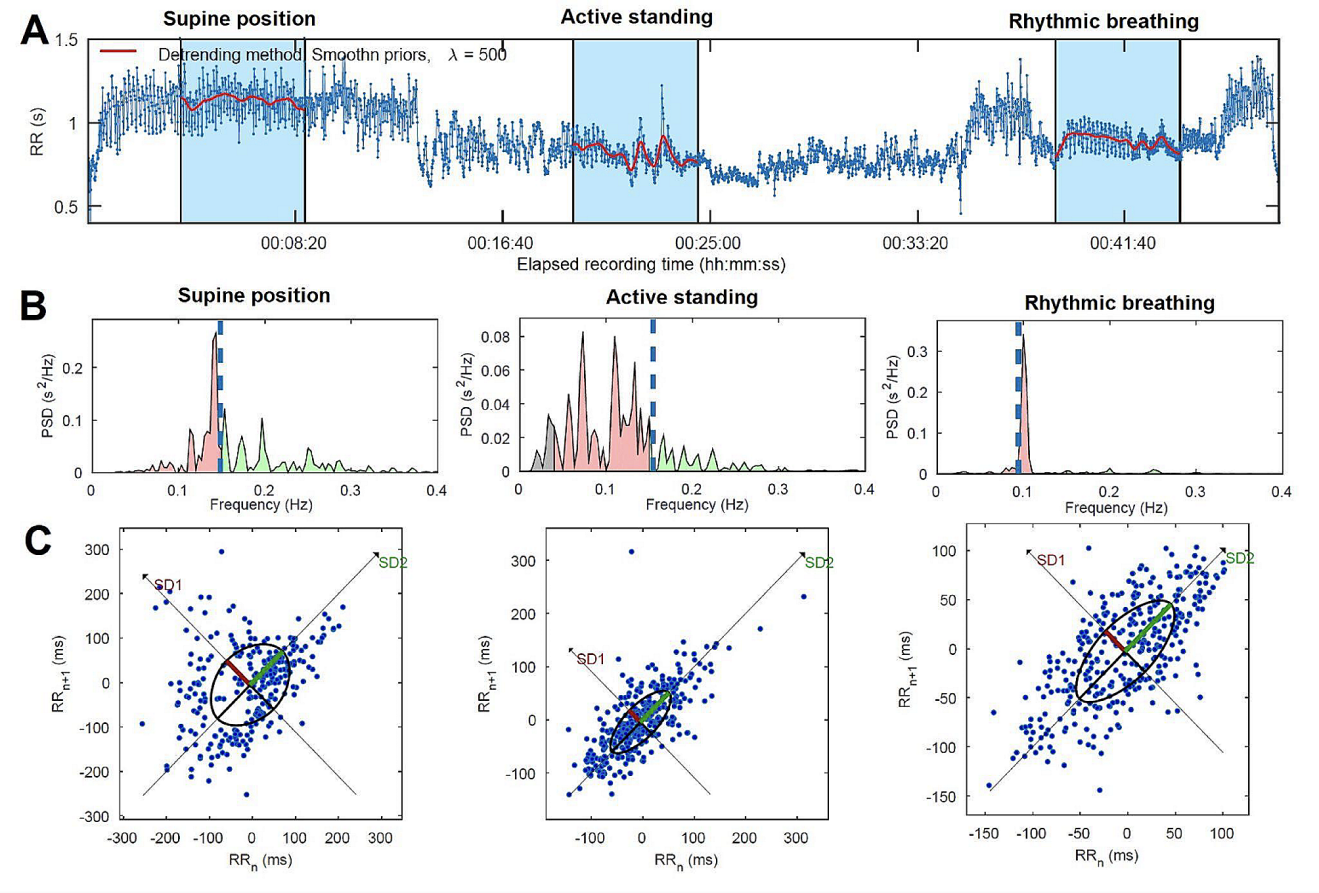
Example of HRV analysis from one patient with BC. Caption: (**A**) RR interval time series during the study protocol. (**B**) Power spectrum density plot (PSD) obtained with the fast Fourier transform (FFT) based Welch’s periodogram method. (**C**) Poincaré plot analysis with the ellipse fitting procedure. SD1 and SD2 are the standard deviations perpendicular to and along the identity line (i.e., RR_n_ = RR_*n*+1_), respectively. Abbreviations: Hz, Hertz; ms, Milliseconds; RR_n_, RR intervals as the x-axis; RR_*n*+1_, RR intervals delayed by one heartbeat on the y-axis

The HRV protocol was conducted following the guidelines outlined by Laborde et al. (2017), to assess tonic HRV (supine position) and phasic HRV (active standing and rhythmic breathing). This evaluation gauges system’s reactivity to different physiological challenges, reflecting changes in HRV (supine position vs. active standing or supine position vs. rhythmic breathing) [15]. The ECG was initially recorded in a supine position for 10 min, followed by two consecutive physiological maneuvers: active standing and rhythmic breathing (at 0.1 Hz and 0.25 Hz), each lasting 10 min (Fig. 2).

Artifacts and ectopic beats were visually identified to eliminate artifacts and RR intervals stemming from ectopic beats. The HRV time series were then derived exclusively from complexes of sinus node origin (NN intervals) [16]. From each condition (supine position, active standing, and controlled breathing), time series of 300-second intervals were chosen by selecting stable segments after the initial 180 s in each position (Fig. 2A). To prevent introducing variations in the estimation of scaling indexes (described below), all series were standardized to this specific number of intervals, covering approximately 5 min. This practice avoids potential discrepancies that may arise from analyzing series with different numbers of NN intervals [17]. For each NN time series, a HRV analysis was conducted following international recommendations and established methods [18]. The calculated time-domain HRV indexes included mean NN (mean value of all NN intervals), SDNN (standard deviation of all NN intervals), and pNN50 (percentage of successive NN intervals with differences greater than 50 ms).

To estimate frequency domain indexes, each NN time series was resampled using a linear interpolation method at three samples per second. The power spectrum density (PSD) was then obtained utilizing Welch’s periodogram (Fig. 2B). Mean spectral power was calculated for the low-frequency band (LF, 0.04 to 0.15 Hz), associated with both sympathetic and parasympathetic activity, and the high-frequency band (HF, 0.15 to 0.4 Hz), a reliable parameter linked to parasympathetic activity [19]. LF and HF values were transformed into normalized units (nu).

Finally, for each NN time series, the Poincaré plot was generated by plotting a scattergram of the RR intervals as the x-axis (RR_n_) and the RR intervals delayed by one heartbeat on the y-axis (RR_*n*+1_) (Fig. 2C). Subsequently, an ellipse was fitted to the Poincaré plot points to measure the standard deviation (SD) of the points along the identity line (i.e., RR_n_ = RR_*n*+1_). This SD corresponds to the length of the ellipse’s long axis (SD2) and reflects slow changes contributing to increased long-term variability, potentially associated with sympathetic activity [21]. Additionally, the length of the ellipse’s short axis was calculated as the SD of points perpendicular to the identity line (SD1). This value reflects fast (beat-to-beat) changes in heart rate, potentially associated with parasympathetic activity [20].

For all HRV indexes obtained in this study, we calculated the magnitude of change (Δ) by determining the difference between values in the supine position and those during active standing, as well as values in the supine position and controlled breathing. The estimation of HRV indexes was performed using Kubios HRV in accordance with the guidelines for HRV analysis [16].

### Echocardiographic study

All patients underwent at least two echocardiographic assessments: one at baseline before chemotherapy and another after three months. Evaluations were conducted using a Siemens-Acuson SC2000 echocardiogram, featuring a 4 MHz phased array transducer with M-mode, color Doppler, and 3D imaging. The same vendor and cardiologist conducted and interpreted all studies, respectively. Exclusion criteria included age < 18, male gender, non-sinus rhythm during echocardiographic exam, uncontrolled hypertension, or current treatment with β-blockers, baseline LV systolic dysfunction (LVEF < 53%), history of cardiac disease (i.e., myocardial infarction, myocarditis, severe valve diseases, atrial fibrillation, atrial flutter), prior Anthracycline therapy, concurrent Dexrazoxane therapy, or poor echocardiographic image quality. These conditions were excluded from the study due to their association with autonomic dysfunction and cardiovascular disease. Such comorbidities have the potential to confound the specific effects of oncological factors on HRV.

Echocardiograms assessed left and right cavity diameters, relative wall thickness, LV mass, and systolic and diastolic function. This comprehensive evaluation included 2D and 3D LVEF, LV global longitudinal strain (LV GLS), circumferential (LV GCS) and radial (LV GRS) strains. Left atrial global strain (LAS) in the four-chamber view was determined. The endocardial border was manually traced after detecting and marking fiducial landmarks, followed by adjusting regions of interest. Subsequently, a composite LA longitudinal strain curve throughout the cardiac cycle was generated. LA function calculations, evaluating conduit (LASc), reservoir (LASr), and pump (LASp) functions according to definitions from prior studies [21].

A significant LASr reduction was defined as either: (1) LASr value below the normal threshold of 35% as per current literature, or (2) LAS relative reduction > 10% during follow-up, aligned with the clinically significant reduction definition of LV GLS [22, 23]. Patients with a baseline LASr < 35% were categorized as showing significant LAS reduction only if a > 10% relative reduction occurred during follow-up.

Diastolic function parameters, including E/A mitral ratio, E/e’ ratio, early transmitral flow velocity (E), late atrial contraction (A) velocity, deceleration time (DT), and early diastolic mitral annular velocity (medial and lateral e’), were measured in the apical 4-chamber view. The peak E/e’ ratio was calculated from the average of at least 3 cardiac cycles. Diastolic function was categorized into four groups, as per guidelines: normal diastolic function, impaired relaxation, and pseudonormal and restrictive patterns [24–26].

### Statistical analysis

The Kolmogorov-Smirnov test assessed normal distribution of continues variables. If significant (*p* < 0.05), variables were log-transformed (Log10). Results, reported as mean ± SD, were compared using Student’s t-test or repeated analyses of variance between subjects with and without cardiotoxicity in supine position, active standing, and controlled breathing. Nominal variables, presented as absolute values (percentage), were compared using the chi-squared test or Fisher’s exact test. Pearson’s correlation analyses were performed to explore the potential association between the echocardiographic parameters and HRV index.

**Fig. 3:**
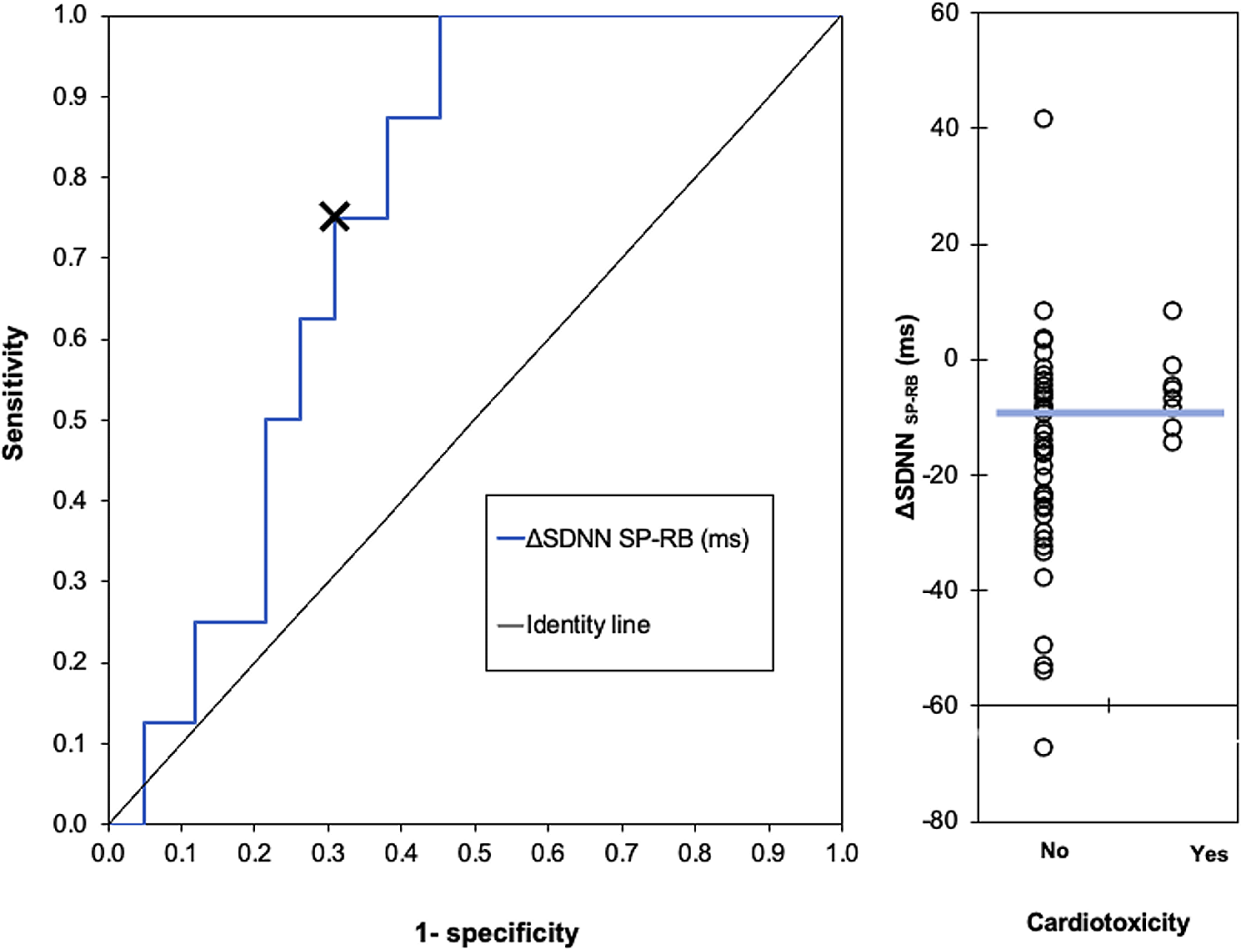
ROC curve analysis of ΔSDNN_SP−RB_. Caption: The best cut-off point (indicated by symbol ‘x’) was determined according to the shortest orthogonal distance from each point to the optimum value (0,1). Abbreviations: ms, Milliseconds; SDNN, Standard deviation of all normal RR intervals; ΔSDNN_SP−RB_, Magnitude of change in SDNN between supine position and rhythmic breathing

Receiver-operator-characteristic (ROC) curve analysis determined the best cut-off point for each Δ HRV index. The cut-off values were based on the shortest orthogonal distance from each curve point to the optimal point (Fig. 3). This approach was employed to report sensitivity and specificity. Statistical analysis was performed using SPSS version 21.0 (IBM Corp., Armonk, NY, USA), with *p* < 0.05 considered statistically significant.

## Results

### Study population and echocardiographic parameters

Fifty patients had comprehensive assessments with baseline and follow-up TTE, HRV studies, and blood pressure (BP) measurements (Fig. 1). The average age was 50 ± 8 years, and reported comorbidities included diabetes (*n* = 9), hypertension (*n* = 17), and dyslipidemia (*n* = 11).

Table 1 shows anthropometric and echocardiographic parameters at baseline and three-month follow-up. Compared to baseline, the three-month follow-up revealed significant changes, including weight loss (*p* = 0.034), decreased BMI (*p* = 0.037), and increased systolic BP (*p* = 0.045). Echocardiography findings at the follow-up revealed smaller LV GLS (*p* = 0.003), LV GCS (*p* < 0.001), LV GRS (*p* = 0.046), LASp (*p* = 0.019), RAS (*p* = 0.031), and RASr (*p* = 0.041).

**Table 1:** Demographic and echocardiographic parameters between baseline and three-month follow-up in 50 BC-patients treated with chemotherapy

	Baseline	3-month follow-up	*P* value
Age (years)	50 ± 8	50 ± 8	0.32
Weight (kg)	67.8 ± 8.8	66.42 ± 9.52	0.034
Body mass index (kg/m^2^)	28.3 ± 3.85	27.76 ± 3.89	0.037
Systolic Blood Pressure (mmHg)	114 ± 11	118 ± 12	0.045
Diastolic Blood Pressure (mmHg)	69 ± 8	71 ± 8	0.17
LVEF 3D (%)	63.53 ± 6.71	62.9 ± 6.41	0.50
LV EDV 3D (ml)	78.66 ± 18.26	77.54 ± 15.76	0.61
LV ESV 3D (ml)	28.74 ± 11.97	29.17 ± 9.53	0.78
LV GLS (%)	− 23.93 ± 3.26	− 22.69 ± 2.58	0.003
LV GCS (%)	− 30.78 ± 3.99	− 28.25 ± 4.24	< 0.001
LV GRS (%)	46.33 ± 10.62	42.61 ± 11.05	0.046
LAS (%)	60.23 ± 22.3	53.52 ± 14.43	0.052
LASr (%)	59.05 ± 22.01	53.57 ± 14.12	0.11
LASp (%)	26.11 ± 10.76	21.9 ± 8.67	0.019
LASc (%)	34.5 ± 17.23	31.67 ± 10.53	0.28
RAS (%)	65.68 ± 22.1	57.45 ± 17.48	0.031
RASr (%)	64.85 ± 21.96	56.92 ± 17.38	0.041
RASp (%)	27.18 ± 13.96	24.8 ± 9.73	0.24
RASc (%)	37.63 ± 14.29	32.12 ± 13.82	0.075
LV MD	51.44 ± 11.21	49.97 ± 9.98	0.27

Abbreviations: LAS: Left atrial strain; LASc: Left atrial strain-conduit phase; LASp: Left atrial strain-pump phase; LASr: Left atrial strain-reservoir phase; LV EDV 3D: Three-Dimensional left ventricular end-diastolic volume; LVEF 3D: Three-Dimensional left ventricular ejection fraction; LV ESV 3D: Three-Dimensional left ventricular end-systolic volume; LV GCS: Left ventricular global circumferential strain; LV GLS: Left ventricular global longitudinal strain; LV GRS: Left ventricular global radial strain; LV MD: Left ventricular mechanical dyssynchrony; RAS: Right atrial strain; RASc: Right atrial strain-conduit phase; RASp: Right atrial strain-pump phase; RASr: Right atrial strain-reservoir phase

### HRV indexes response to physiological stimulus before and after chemotherapy

**Fig. 4:**
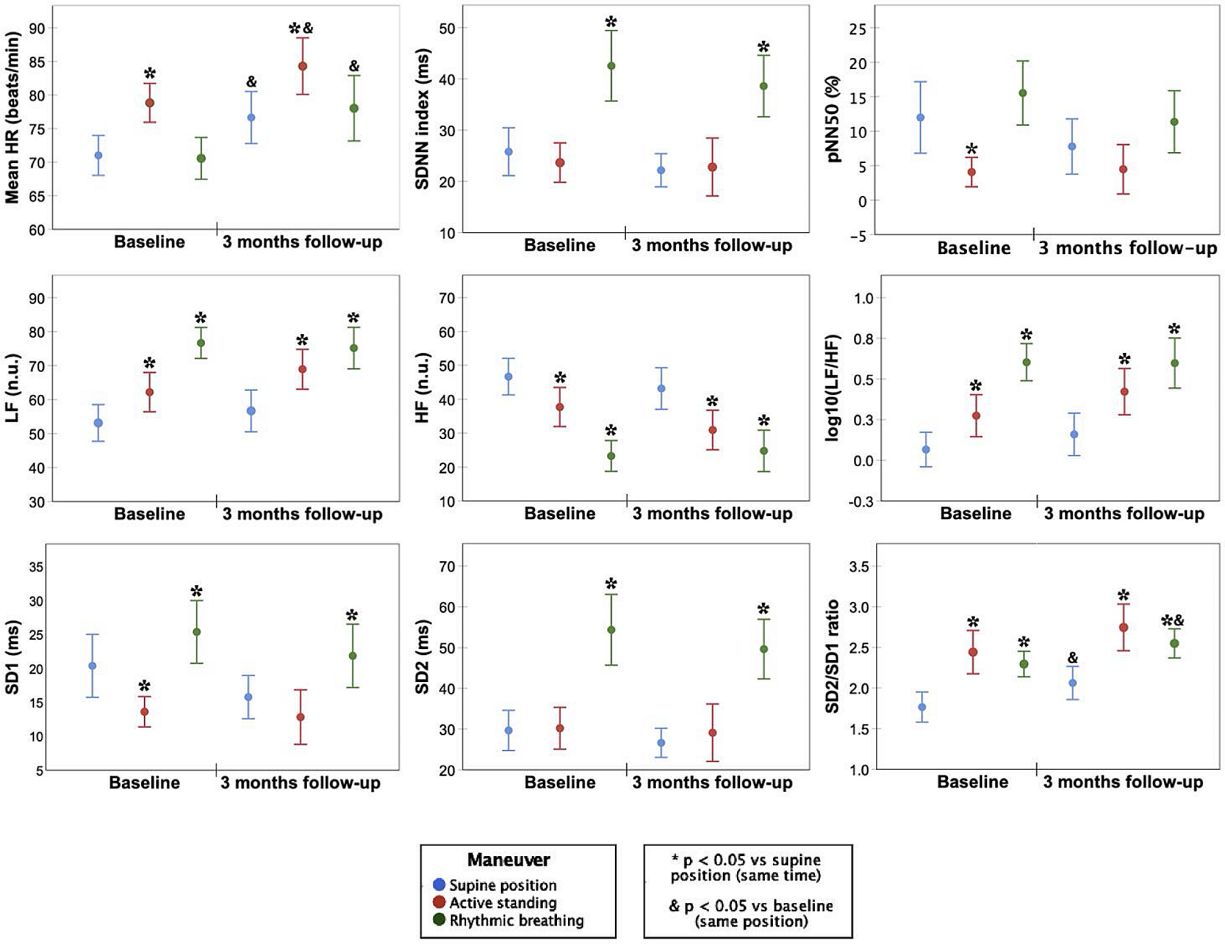
HRV-indexes of 50 BC-patients before and after three-months of chemotherapy. Significant changes between the maneuvers compared to the supine position in the same time period are marked with an asterisk (*). Significant changes in the same position between the two time periods (baseline vs. 3 months follow-up) are marked with an ampersand (&). Abbreviations: HF, High Frequency: Power spectrum in the frequency range of 0.15–0.4; HR, Heart rate; LF, Low Frequency: Power spectrum in the frequency range of 0.04–0.15; log10(LF/HF), Logarithm from ratio of LF to HF power; pNN50, Proportion of successive NN interval differences larger than 50 ms; SD1, Poincaré plot standard deviation perpendicular the line of identity; SD2, Poincaré plot standard deviation along the line of identity; SD1/SD2, Ratio of SD1 to SD2SDNN, Standard deviation of NN intervals

Figure 4 shows the cardiac autonomic activity evaluation results before and after three-months of chemotherapy in supine position, active standing, and with rhythmic breathing. After three-months of chemotherapy, mean HR increased significantly in all maneuvers, and SD2/SD1 ratio increased in the supine position and with controlled breathing.

Time domain parameters showed increased mean HR during active standing and increased SDNN during controlled breathing, and before and after chemotherapy. However, pNN50 only decreased before chemotherapy in response to active standing. In the frequency domain parameters, LF increased, HF decreased, and the LF/HF ratio increased with both stimuli (active standing and controlled breathing) and before and after chemotherapy. Poincaré plot indexes showed SD1 decrease with active standing only before chemotherapy and increase during controlled breathing (before and after chemotherapy). Simultaneously, SD2 increased during controlled breathing (before and after chemotherapy), and the SD1/SD2 ratio increased in response to both stimuli active standing and controlled breathing, before and after chemotherapy.

Pearson’s correlation analyses were performed to explore the potential association between the echocardiographic parameters and HRV index (Tables S1 and S2 in the Supplementary material). In the assessment before chemotherapy (Table S1), LASc had a significant association with LF, HF, and LF/HF (during active standing), while during rhythmic breathing, LF/HF had a significant association with LAS, LASr, and LASc. Also, SD2/SD1 had a significant association with LASr. In the assessment after three months of chemotherapy (Table S2), LASr had a significant correlation with the mean heart rate (during the supine position, and LASp had a significant correlation with HF, LF/HF, and SD2/SD1 (during active standing).

### Cardiotoxicity after 3 months of chemotherapy

**Fig. 5:**
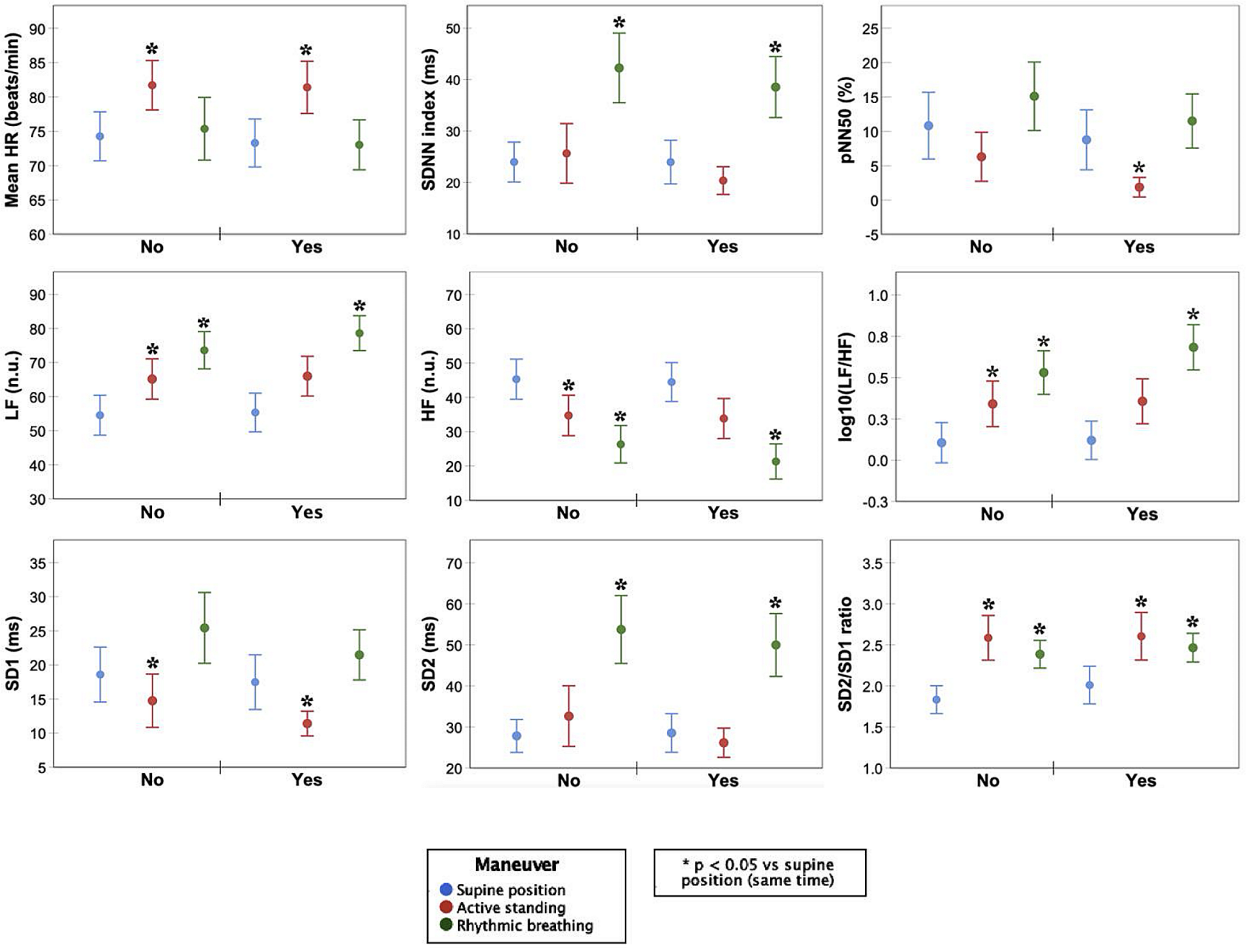
HRV-indexes before chemotherapy in 23 BC-patients who developed cardiotoxicity by any criteria. Significant changes (*p* < 0.05) between the maneuvers compared to the supine position in the same group are marked with an asterisk (*). Abbreviations as before

**Fig. 6:**
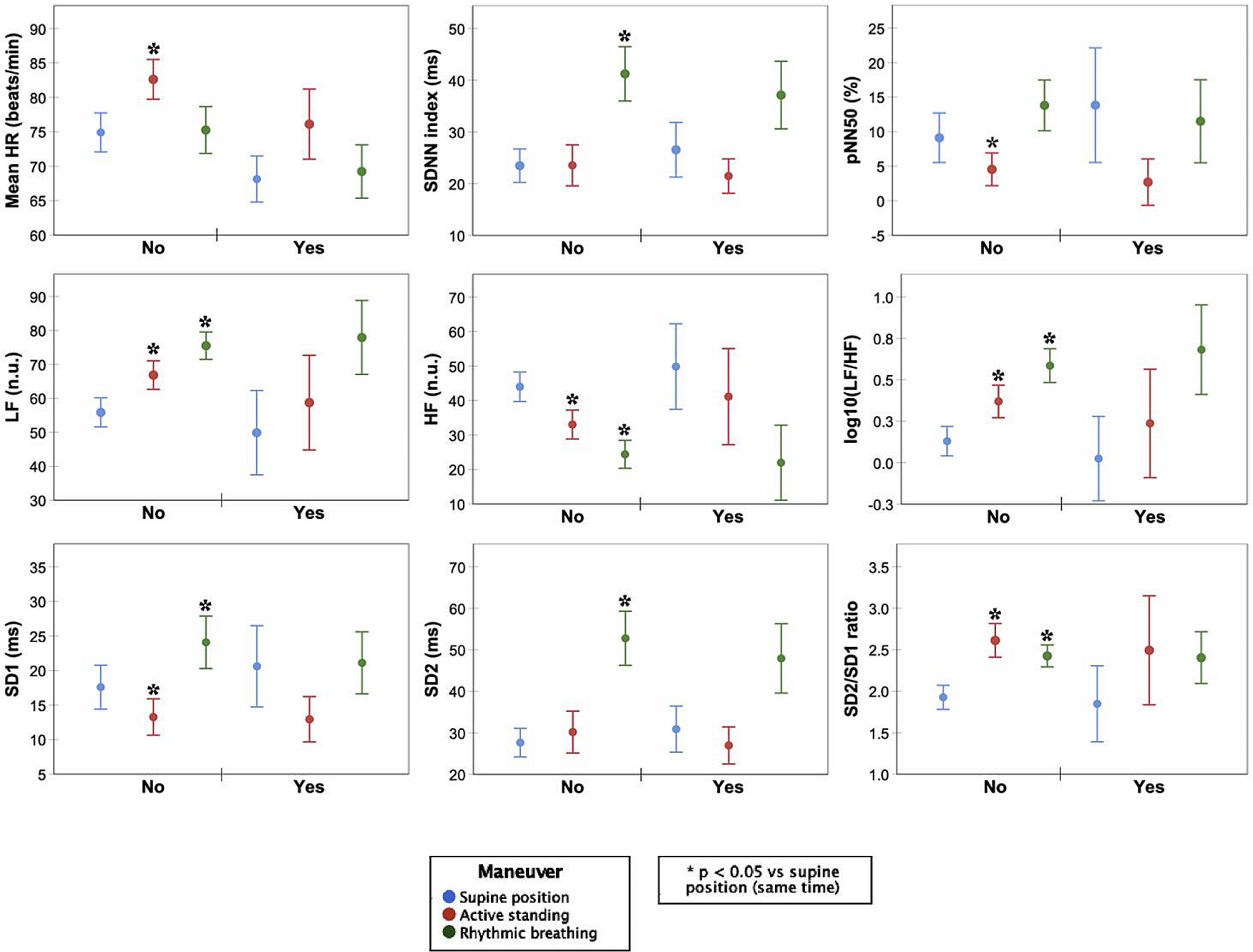
HRV-indexes before chemotherapy in 8 BC-patients with cardiotoxicity based on echocardiography. Significant changes (*p* < 0.05) between the maneuvers compared to the supine position in the same time period are marked with an asterisk (*). Abbreviations as before

**Fig. 7:**
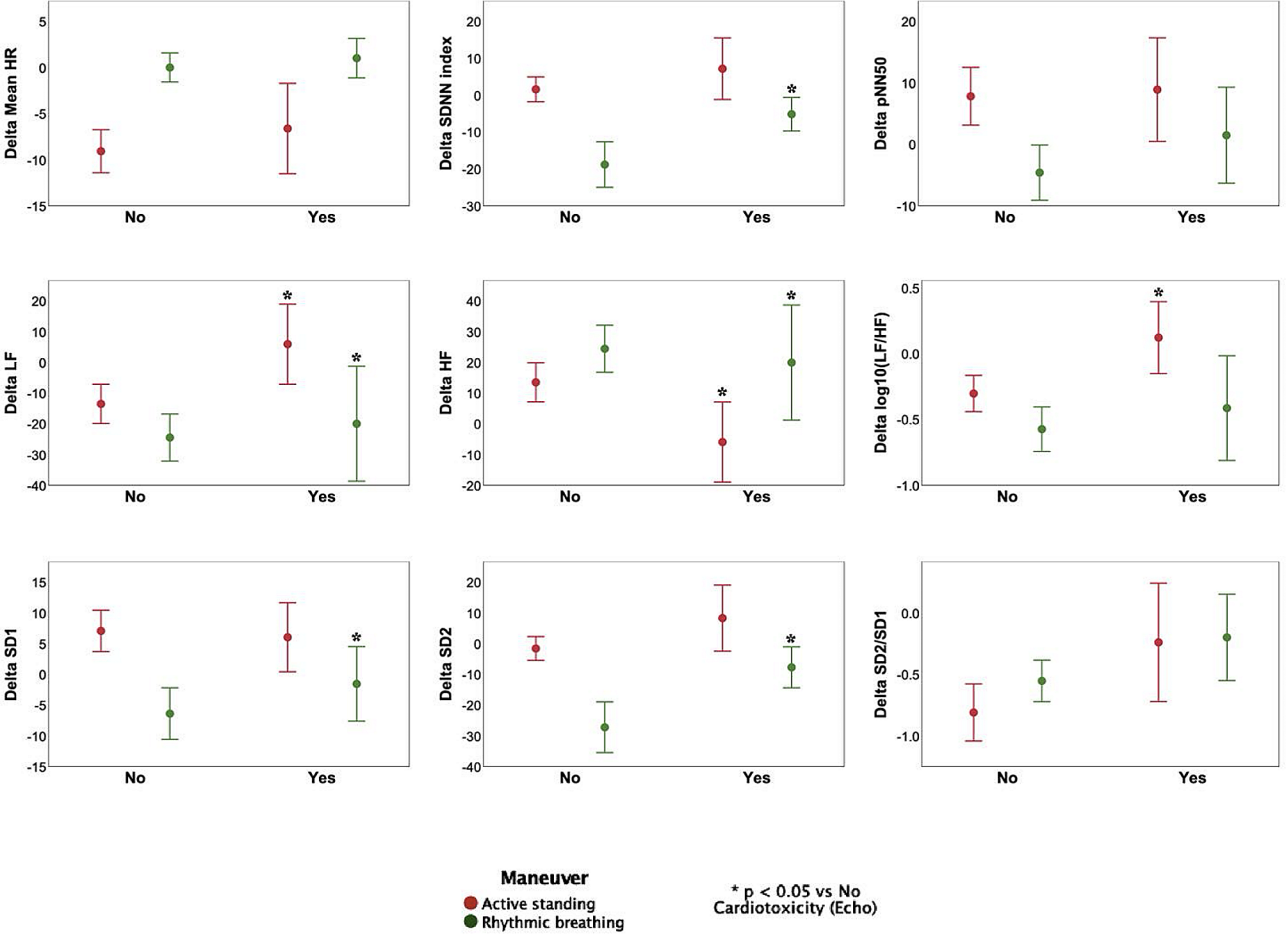
ΔHRV-indexes in 8 BC-patients with cardiotoxicity based on echocardiography in response to active standing and rhythmic breathing. Significant changes (*p* < 0.05) between cardiotoxicity and non-cardiotoxicity groups in a maneuver are marked with an asterisk (*). Abbreviations as before

Twenty-three patients fulfilled echocardiographic and troponin cardiotoxicity criteria or both (Fig. 1), while 27 patients did not. Table 2 compares anthropometric and echocardiographic variables between groups that fulfilled cardiotoxicity criteria (*n* = 23). Notably, only LASc during baseline was significantly increased in the cardiotoxicity group compared to the group without cardiotoxicity. Compared to before chemotherapy, within the cardiotoxicity group the systolic blood pressure was increased, while the LV GRS and LASp decreased after three months of chemotherapy. In the group without cardiotoxicity, the body mass index, RAS and RASr decreased after three months of chemotherapy. Figure 5 shows HRV assessments before chemotherapy between those who developed cardiotoxicity by any criteria (*n* = 23) and those who did not (*n* = 27). No significant changes were observed in parameters induced by maneuvers between the groups. Both those who developed cardiotoxicity and those who did not exhibited similar HR, SDNN, SD1, SD2, and the SD2/SD1 ratio responses in both maneuvers. Both groups showed similar controlled breathing responses in LF, HF, and LF/HF. However, during active standing, pNN50 decreased only in the cardiotoxicity group, and LF, HF, and LF/HF changed only in the non-cardiotoxicity group, with no changes observed in the cardiotoxicity group. Table 3 compares anthropometric and echocardiographic parameters between patients fulfilling echocardiographic cardiotoxicity criteria (*n* = 8) and those who did not (*N* = 42). There were no significant differences between groups before chemotherapy. After three months of chemotherapy, the group with cardiotoxicity had decreased LVEF 3D and LV GRS, while LV GLS increased. Compared to before chemotherapy, the group with cardiotoxicity had increased systolic blood pressure, LV GLS and LV GCS, while LVEF 3D was decreased. Meanwhile, the group without cardiotoxicity had increased LV GCS and decreased RAS, RASr, and RASc. Figure 6 compares HRV assessments before chemotherapy in those with echocardiographic-defined cardiotoxicity (*N* = 8) and those without (*N* = 42). No significant changes in parameters induced by maneuvers were observed between the groups, except for a noticeable difference in the orthostatic challenge response. Patients without cardiotoxicity had significant active standing HRV index changes (except SDNN and SD2). Those *with* cardiotoxicity showed no significant index changes. Similarly, rhythmic breathing led to significant HRV index changes in non-cardiotoxicity patients, while no changes occurred in cardiotoxicity patients. Figure 7 depicts Δ in HRV indexes for active standing and rhythmic breathing. Patients with cardiotoxicity exhibited smaller Δ in LF, HF, and LF/HF during active standing, and in SDNN, SD1, and SD2 during controlled breathing compared to those without cardiotoxicity. Table 4 presents ROC curve analysis for Δ in HRV indexes. The smaller changes in four indexes (ΔSDNN, ΔpNN50, ΔSD1, and ΔSD2) observed during controlled breathing could serve as predictors of post-chemotherapy cardiotoxicity.

**Table 2:** Demographic and echocardiographic parameters before and after three months of chemotherapy of patients who met echocardiographic, troponins, or both criteria for cardiotoxicity

Variable	Cardiotoxicity (Echocardiography, Troponins, Echo + Troponins)			
	Yes (*N* = 23)		No (*N* = 27)	
	Before	After	Before	After
Age (years)	49 ± 7	49 ± 7	50 ± 9	50 ± 9
Weight (kg)	67.2 ± 8.5	66.2 ± 8.3	68.3 ± 9.2	66.6 ± 10.6
Body mass index (kg/m^2^)	27.89 ± 3.89	27.53 ± 3.61	28.65 ± 3.85	27.94 ± 4.17^&^
Systolic Blood Pressure (mmHg)	111 ± 9	117 ± 11^&^	116 ± 12	119 ± 13
Diastolic Blood Pressure (mmHg)	70 ± 8	72 ± 9	69 ± 8	71 ± 8
LVEF 3D (%)	65.0 ± 8.3	62.7 ± 8.3	62.3 ± 4.8	63.1 ± 4.4
LV EDV 3D (ml)	78.96 ± 22.06	80.04 ± 18.14	78.4 ± 14.71	75.4 ± 13.39
LV ESV 3D (ml)	27.78 ± 16.18	30.51 ± 12.20	29.56 ± 6.86	28.02 ± 6.51
LV GLS (%)	-24.31 ± 4.35	-22.23 ± 3.18	-23.62 ± 1.96	-23.08 ± 1.92
LV GCS (%)	-30.63 ± 4.44	-27.26 ± 3.79	-30.9 ± 3.65	-29.10 ± 4.48
LV GRS (%)	48.16 ± 13.46	41.06 ± 9.06^&^	44.78 ± 7.35	43.93 ± 12.52
LAS (%)	65.98 ± 25.27	53.93 ± 14.93	55.33 ± 18.50	53.17 ± 14.26
LASr (%)	63.75 ± 25.04	54.11 ± 14.37	55.05 ± 18.60	53.11 ± 14.16
LASp (%)	26.66 ± 11.88	20.32 ± 8.30^&^	25.64 ± 9.92	23.25 ± 8.90
LASc (%)	40.46 ± 19.99	33.79 ± 10.99	29.41 ± 12.77*	29.86 ± 9.96
RAS (%)	61.94 ± 21.47	57.86 ± 17.63	68.87 ± 22.53	57.1 ± 17.69^&^
RASr (%)	60.60 ± 21.02	57.47 ± 17.63	68.47 ± 22.48	56.46 ± 17.48^&^
RASp (%)	23.25 ± 9.6	23.88 ± 10.47	30.53 ± 16.24	25.59 ± 9.18
RASc (%)	37.27 ± 14.45	33.58 ± 14.42	37.94 ± 14.42	30.87 ± 13.44
LV MD	50.02 ± 13.07	48.25 ± 10.33	52.65 ± 9.43	51.44 ± 9.62

* *p* < 0.05 compared to patients with cardiotoxicity (same time)

^&^*p* < 0.05 compared to before chemotherapy (within same group)

Abbreviations: As in Table 1

**Table 3:** Demographic and echocardiographic parameters before and after three months of chemotherapy of patients who met echocardiographic criteria for cardiotoxicity

Variable	Cardiotoxicity (Echocardiography)			
	Yes (*N* = 8)		No (*N* = 42)	
	Before	After	Before	After
Age (years)	50 ± 6	50 ± 6	49 ± 9	49 ± 9
Weight (kg)	67.5 ± 10.5	65.17 ± 11.5	68.0 ± 8.6	66.8 ± 9.2
Body mass index (kg/m^2^)	29.07 ± 4.54	28.46 ± 5.20	28.14 ± 3.70	27.65 ± 3.60
Systolic Blood Pressure (mmHg)	109 ± 8	119 ± 11^&^	116 ± 11	117 ± 13
Diastolic Blood Pressure (mmHg)	68 ± 7	73 ± 10	70 ± 8	71 ± 8
LVEF 3D (%)	63.44 ± 7.77	56.91 ± 11.28^&^	63.58 ± 6.56	64.09 ± 4.29*
LV EDV 3D (ml)	82.25 ± 25.14	86.51 ± 27.13	78.26 ± 18.40	75.26 ± 12.38
LV ESV 3D (ml)	30.96 ± 12.97	38.54 ± 17.74	28.50 ± 12.28	27.15 ± 5.87*
LV GLS (%)	-24.39 ± 4.24	-19.93 ± 3.89^&^	-23.76 ± 3.10	-23.12 ± 1.94*
LV GCS (%)	-31.35 ± 5.24	-25.44 ± 4.37^&^	-30.78 ± 3.73	-28.91 ± 4.04*^&^
LV GRS (%)	41.58 ± 10.4	33.71 ± 8.36	47.68 ± 11.18	44.73 ± 10.80*
LAS (%)	60.95 ± 22.06	51.14 ± 22.91	60.25 ± 22.25	53.82 ± 12.69
LASr (%)	60.51 ± 22.04	50.93 ± 22.68	58.96 ± 21.91	53.91 ± 12.35
LASp (%)	26.83 ± 10.58	20.10 ± 12.42	25.65 ± 10.78	22.33 ± 7.91
LASc (%)	33.68 ± 15.36	30.82 ± 13.93	35.07 ± 17.55	31.58 ± 9.92
RAS (%)	57.86 ± 21.71	59.73 ± 19.60	66.98 ± 21.78	56.70 ± 16.94^&^
RASr (%)	57.63 ± 21.66	59.57 ± 19.65	65.98 ± 21.67	56.12 ± 16.78^&^
RASp (%)	24.37 ± 10.16	23.12 ± 13.40	27.75 ± 14.37	25.16 ± 8.85
RASc (%)	33.26 ± 12.53	36.46 ± 13.79	38.20 ± 14.32	30.93 ± 13.66^&^
LV MD	55.35 ± 15.94	53.41 ± 11.38	50.7 ± 10.16	49.31 ± 6.70

* *p* < 0.05 compared to patients with cardiotoxicity (same time)

^&^*p* < 0.05 compared to before chemotherapy (within same group)

Abbreviations: As in Table 1

**Table 4:** ROC curve analysis of the magnitude of change Δ in HRV indexes

	AUC (95% CI)	*P*-value ^a^	Best cut-off value	Sensitivity (%)	Specificity (%)
**Supine position vs. Active standing**					
ΔMean HR	0.55	0.63	≤ -3	50	81
ΔSDNN	0.65	0.20	≤ 0.30	88	52
ΔpNN50	0.57	0.54	≤ 2.26	63	62
ΔLF	0.71	0.064	≤ -1.29	63	67
ΔHF	0.71	0.060	≤ 1.10	63	67
Δlog10 (LF/HF)	0.71	0.068	≤ -0.021	63	67
ΔSD1	0.47	0.81	≤ 3.83	50	55
ΔSD2	0.68	0.12	≤ 2.45	63	71
ΔSD2/SD1	0.66	0.15	≤ -0.34	63	74
**Supine position vs. Rhythmic Breathing**					
ΔMean HR	0.44	0.60	≤ 0.28	63	43
ΔSDNN	0.75	0.026	≤ -8.44	75	69
ΔpNN50	0.73	0.046	≤ -1.64	88	55
ΔLF	0.57	0.53	≤ -13.85	63	74
ΔHF	0.57	0.53	≤ 13.28	63	74
Δlog10 (LF/HF)	0.58	0.48	≤ -0.30	63	74
ΔSD1	0.74	0.030	≤ -0.66	63	76
ΔSD2	0.73	0.044	≤ -17.40	88	64
ΔSD2/SD1	0.66	0.15	≤ -0.42	63	64

^a^ Null hypothesis is that area under the curve = 0.5. Abbreviations: HF, Power spectrum in the frequency range of 0.15–0.4; HR, Heart rate; LF, Power spectrum in the frequency range of 0.04–0.15; log10(LF/HF), Logarithm from ratio of LF to HF power; pNN50, Proportion of successive NN interval differences larger than 50 ms; SD1, Poincaré plot standard deviation perpendicular the line of identity; SD2, Poincaré plot standard deviation along the line of identity; SD1/SD2, Ratio of SD1 to SD2SDNN, Standard deviation of NN intervals

## Discussion

### Comparison of TTE results versus previous studies

Early detection of chemotherapy-related cardiotoxicity is crucial for preventing adverse effects and improving patient care. TTE is the first-line cardiac imaging technique for this purpose; according to ESC guidelines, cardiac dysfunction diagnosis relies on reduced LVEF and GLS [8]. Some investigators propose new echocardiographic parameters for early disfunction prediction. Bennet, et al. conducted a systematic review to assess the Tei-index evidence for detecting LV systolic dysfunction. They suggested it might be useful in evaluating subclinical anthracycline-related cardiotoxicity. However, inconsistent findings warrant further evaluation [27].

In our population, after three-months of chemotherapy, LV GLS, GCS, and GRS were notably reduced. Research indicates that reduced GLS can reveal subclinical systolic LV dysfunction at an early stage when LVEF may be normal [28]. This reduction offers insights into myocardial disease and correlates with worse outcomes. GCS and GRS have lower reproducibility; thus, their utility may be less than that of GLS [29].

Regarding LA strains, multiple studies have explored reservoir and pump phase associations with filling pressures, relying on both LV systolic and diastolic functions and emphasizing LVEF. In our population, LASp was decreased. Clinically, this may indicate atrial myopathy or stunning post-atrial arrhythmia rather than high filling pressures [30]. The reduced RASr in our population serves as a non-invasive means of detecting elevated RA pressure often seen in heart failure patients [31]. Stratifying our population based on any cardiotoxicity criteria revealed an elevated LASc. Though this phase contributes to LV stroke volume, its elevation under certain conditions, such as resistance to LV filling, may indicate deterioration of diastolic function and heart failure [32]. However, subjects meeting only echocardiographic criteria showed no group differences. This highlights the importance of biomarkers in identifying additional cases of CTRCD not fully detected through imaging.

### Comparison of HRV results versus previous studies

HRV analysis assesses cardiac function based on the impact of sympathetic and parasympathetic systems on BP and vascular tone regulation [33]. In healthy patients, supine position shows similar parasympathetic and sympathetic responses. With an orthostatic stimulus, the parasympathetic response decreases while the sympathetic response has a major impact. The sympathetic response reduces HRV with a predominance of LF over HF oscillations, while the parasympathetic response is characterized by HF oscillations [34, 35]. Lakoski et al. observed SNS overdrive and reduced PSNS efficacy related to CTRCD in BC patients [6]. Feng et al. reported lower root mean square of successive differences (RMSSD), SDNN and DC values in *HER2 +* BC treated with Trastuzumab [11]. Tjeerdsma et al. found that BC patients on anthracycline-based chemotherapy exhibited nearly 85% abnormal HRV, specifically in PSNS parameters like RMSSD and pNN50 [36]. That is, PSNS dysfunction is the primary autonomic impairment in BC patients undergoing anthracycline and trastuzumab chemotherapy.

Our HRV analysis during controlled maneuvers revealed preserved cardiac autonomic modulation response to physiological stimuli on active standing and controlled breathing after three months of chemotherapy (Fig. 4). This normal response was characterized by increased sympathetic activity and decreased parasympathetic activity during active standing evident in HR, LF, HF, LF/HF, SD1, SD2, and SD1/SD2 (Fig. 4) [37]. Controlled breathing response showed increased cardiorespiratory coupling with higher HRV fluctuation amplitudes (SDNN, SD1, SD2) and a significant rise in mean power around slow fluctuations (LF, SD1) due to increased coupling around the 0.1 Hz rhythmic pattern [38]. This dominance of slow large-amplitude fluctuations induced by rhythmic breathing reflects higher LF/HF and SD2/SD1 ratio values [39]. Interestingly, the preserved response to physiologic stimuli in all HRV indexes was found with a consistent increase in mean HR post-chemotherapy. Considering the HR as a controlled variable adjusted by feedback mechanisms to maintain cardiovascular system stability in response to stimuli, a consistent increase in mean HR suggests a shift in the set-point of such regulatory mechanisms to faster HR after only three-months of chemotherapy [40, 41]. While HR stays within normal range, a persistent increase in mean HR may not be innocuous, since an elevated resting HR is a recognized indicator of risk in numerous cardiovascular outcomes [42, 43].

When we compared changes in response to physiological stimuli (active standing and rhythmic breathing) before chemotherapy in those developing cardiotoxicity after 3 months, we observed a smaller response in LF, HF, and LF/HF indexes to active standing and in SDNN, LF, HF, SD1, and SD2 to rhythmic breathing (Fig. 7). This suggests a decreased adaptive response to physiological stimuli even before chemotherapy only in those who developed cardiotoxicity after three-months of treatment. The diminished response to active standing aligns with a lower sympathetic increase and parasympathetic decrease, resulting in less sympathetic autonomic modulation during orthostatic stress. The smaller response to rhythmic breathing suggests weaker cardiorespiratory coupling in patients who might develop cardiotoxicity. Lower autonomic reactivity in BC patients undergoing chemotherapy compared to healthy individuals has been documented with baseline HRV and Ewing’s cardiac autonomic reactivity tests [44]. Our findings indicate that a pre-existing lower autonomic reactivity before chemotherapy could predict early cardiotoxicity. HRV indexes during physiological stimuli could potentially act as complementary non-invasive biomarkers for assessing cardiotoxicity risk.

### Study limitations

The study’s small sample size may limit generalizing our findings to a broader BC patient population. A larger sample size could yield more robust results. Our study has a relatively short follow-up period (three-months), and cardiotoxicity may develop over a more extended period. A longer follow-up could provide a more comprehensive understanding of cardiac responses. In addition, the fact that the study was carried out in a single center might call into question the universal value of its conclusions. Multi-center studies can offer more diverse and generalizable results.

## Conclusions

In conclusion, BC patients’ cardiac autonomic response to physiological stimuli appears to maintain its responsiveness even after three months of chemotherapy with anthracyclines with or without trastuzumab. However, a noteworthy finding is a reduced Δ in response to physiological stimuli before chemotherapy in patients who developed cardiotoxicity (identified by echocardiography) compared to those who did not. Significant LASc differences were also observed between those with and without cardiotoxicity. These HRV indexes may hold promise as non-invasive predictors of early cardiotoxicity.

### Electronic supplementary material

Below is the link to the electronic supplementary material.

Supplementary Material 1

## Data Availability

The datasets used and analysed during the current study are available from the corresponding author on reasonable request.

## Abbreviations

BC: Breast cancer
CTRCD: Cancer therapy-related cardiovascular dysfunction
HF: High Frequency: Power spectrum in the frequency range of 0.15–0.4
HRV: Heart rate variability
LAS: Left atrial strain
LF: Low Frequency: Power spectrum in the frequency range of 0.04–0.15
LV GLS: Left ventricular global longitudinal strain
RAS: Right atrial strain
TTE: Transthoracic echocardiography
