# Supplementary material for: Heart rate variability-based prediction of early cardiotoxicity in breast-cancer patients treated with anthracyclines and trastuzumab
Source: Cardiooncology. 2024 May 29;10:32. doi: 10.1186/s40959-024-00236-y (PMC11134897; doi:10.1186/s40959-024-00236-y)
Supplement: Supplementary file 1 — Supplementary Material 1 [file 40959_2024_236_MOESM1_ESM.docx]

**Heart Rate Variability-Based Prediction of Early Cardiotoxicity in Breast-Cancer Patients Treated with Anthracyclines and Trastuzumab**

**Supplementary material**

**Table S1.** Pearson's correlation coefficients between echocardiographic parameters and HRV indices assessed before chemotherapy in 50 BC patients.

|  | LVGLS (%) | LVGCS (%) | LVGRS (%) | LAS (%) | LASr (%) | LASp (%) | LASc (%) | LVEF 3D (%) |  |
| --- | --- | --- | --- | --- | --- | --- | --- | --- | --- |
| **Supine position** |  |  |  |  |  |  |  |  |  |
| Mean HR (beats/min) | 0.17 | -0.20 | 0.01 | 0.01 | 0.04 | 0.02 | -0.02 | -0.03 |  |
| SDNN index (ms) | -0.05 | 0.16 | 0.02 | -0.01 | 0.01 | -0.11 | 0.03 | 0.05 |  |
| pNN50 (%) | -0.04 | 0.15 | -0.04 | -0.05 | -0.02 | -0.14 | 0.00 | 0.01 |  |
| LF (n.u.) | 0.13 | -0.12 | 0.07 | 0.24 | 0.27 | 0.08 | 0.24 | 0.02 |  |
| HF (n.u.) | -0.13 | 0.11 | -0.07 | -0.24 | -0.26 | -0.08 | -0.24 | -0.02 |  |
| Log LF/HF | 0.13 | -0.11 | 0.06 | 0.24 | 0.26 | 0.08 | 0.24 | 0.03 |  |
| SD1 (ms) | -0.06 | 0.22 | -0.01 | -0.04 | -0.03 | -0.10 | 0.00 | 0.05 |  |
| SD2 (ms) | -0.04 | 0.10 | 0.05 | 0.01 | 0.03 | -0.11 | 0.05 | 0.06 |  |
| SD2/SD1 | 0.15 | -0.16 | 0.06 | 0.18 | 0.21 | 0.05 | 0.16 | -0.01 |  |
| **Active standing** |  |  |  |  |  |  |  |  |  |
| Mean HR (beats/min) | 0.14 | -0.17 | -0.03 | 0.04 | 0.06 | 0.00 | 0.04 | 0.01 |  |
| SDNN index (ms) | -0.02 | 0.18 | 0.01 | 0.00 | 0.01 | -0.01 | -0.02 | 0.06 |  |
| pNN50 (%) | 0.02 | 0.23 | -0.06 | -0.16 | -0.16 | -0.03 | -0.22 | 0.04 |  |
| LF (n.u.) | -0.10 | 0.16 | 0.08 | 0.22 | 0.17 | 0.05 | 0.32* | 0.17 |  |
| HF (n.u.) | 0.10 | -0.16 | -0.08 | -0.22 | -0.17 | -0.05 | -0.32* | -0.17 |  |
| Log LF/HF | -0.15 | 0.15 | 0.09 | 0.21 | 0.15 | 0.07 | 0.29* | 0.19 |  |
| SD1 (ms) | -0.01 | 0.22 | -0.04 | -0.08 | -0.07 | -0.01 | -0.14 | 0.04 |  |
| SD2 (ms) | -0.02 | 0.16 | 0.03 | 0.03 | 0.04 | 0.00 | 0.02 | 0.06 |  |
| SD2/SD1 | -0.03 | 0.02 | -0.05 | 0.10 | 0.09 | -0.07 | 0.18 | 0.05 |  |
| **Rhythmic breathing** |  |  |  |  |  |  |  |  |  |
| Mean HR (beats/min) | 0.11 | -0.19 | 0.00 | 0.05 | 0.07 | 0.03 | 0.04 | 0.01 |  |
| SDNN index (ms) | 0.15 | 0.24 | -0.16 | 0.00 | 0.03 | -0.17 | 0.06 | -0.14 |  |
| pNN50 (%) | 0.14 | 0.19 | -0.11 | -0.04 | -0.02 | -0.19 | 0.03 | -0.13 |  |
| LF (n.u.) | -0.02 | 0.23 | -0.13 | 0.24 | 0.27 | 0.06 | 0.23 | -0.09 |  |
| HF (n.u.) | 0.02 | -0.23 | 0.13 | -0.24 | -0.27 | -0.06 | -0.23 | 0.09 |  |
| Log LF/HF | 0.02 | 0.21 | -0.11 | 0.29* | 0.32* | 0.07 | 0.29* | -0.12 |  |
| SD1 (ms) | 0.12 | 0.20 | -0.14 | -0.07 | -0.05 | -0.16 | -0.02 | -0.07 |  |
| SD2 (ms) | 0.15 | 0.25 | -0.17 | 0.01 | 0.04 | -0.18 | 0.08 | -0.15 |  |
| SD2/SD1 | 0.05 | 0.04 | -0.01 | 0.26 | 0.30* | 0.09 | 0.24 | -0.12 |  |
| * p < 0.05 | | | | | | | | | |

**Table S2.** Pearson's correlation coefficients between echocardiographic parameters and HRV indices three months after chemotherapy.

|  | LVGLS (%) | LVGCS (%) | LVGRS (%) | LAS (%) | LASr (%) | LASp (%) | LASc (%) | LVEF 3D (%) |
| --- | --- | --- | --- | --- | --- | --- | --- | --- |
| **Supine position** |  |  |  |  |  |  |  |  |
| Mean HR (beats/min) | -0.07 | -0.06 | -0.25 | -0.27 | -0.28* | -0.33 | -0.10 | 0.08 |
| SDNN index (ms) | 0.18 | -0.12 | 0.18 | 0.13 | 0.12 | 0.12 | 0.06 | -0.06 |
| pNN50 (%) | 0.20 | -0.07 | 0.02 | 0.02 | 0.01 | 0.13 | -0.09 | -0.17 |
| LF (n.u.) | -0.17 | 0.07 | -0.07 | -0.02 | -0.03 | -0.02 | -0.03 | 0.03 |
| HF (n.u.) | 0.17 | -0.07 | 0.07 | 0.03 | 0.04 | 0.02 | 0.03 | -0.03 |
| Log LF/HF | -0.14 | 0.09 | -0.09 | -0.04 | -0.05 | -0.02 | -0.05 | 0.02 |
| SD1 (ms) | 0.17 | -0.11 | 0.12 | 0.09 | 0.09 | 0.15 | 0.00 | -0.08 |
| SD2 (ms) | 0.18 | -0.13 | 0.20 | 0.15 | 0.14 | 0.11 | 0.10 | -0.06 |
| SD2/SD1 | -0.01 | 0.11 | -0.06 | -0.08 | -0.08 | -0.20 | 0.05 | -0.01 |
| **Active standing** |  |  |  |  |  |  |  |  |
| Mean HR (beats/min) | -0.09 | 0.08 | -0.18 | -0.20 | -0.20 | -0.31 | -0.01 | -0.01 |
| SDNN index (ms) | 0.07 | -0.20 | 0.11 | 0.11 | 0.10 | 0.18 | -0.01 | 0.04 |
| pNN50 (%) | -0.01 | -0.22 | 0.12 | 0.08 | 0.07 | 0.20 | -0.08 | 0.14 |
| LF (n.u.) | -0.05 | 0.00 | -0.13 | -0.13 | -0.12 | -0.35 | 0.12 | -0.03 |
| HF (n.u.) | 0.05 | 0.00 | 0.12 | 0.13 | 0.13 | 0.35* | -0.12 | 0.03 |
| Log LF/HF | -0.01 | 0.07 | -0.17 | -0.14 | -0.14 | -0.34* | 0.09 | -0.06 |
| SD1 (ms) | 0.01 | -0.20 | 0.17 | 0.16 | 0.15 | 0.26 | -0.02 | 0.12 |
| SD2 (ms) | 0.09 | -0.19 | 0.08 | 0.10 | 0.09 | 0.15 | 0.00 | 0.00 |
| SD2/SD1 | 0.04 | 0.15 | -0.25 | -0.23 | -0.24 | -0.34* | -0.04 | -0.12 |
| **Rhythmic breathing** |  |  |  |  |  |  |  |  |
| Mean HR (beats/min) | -0.10 | 0.02 | -0.05 | 0.24 | 0.24 | 0.17 | 0.19 | -0.02 |
| SDNN index (ms) | 0.11 | -0.22 | 0.02 | -0.10 | -0.11 | -0.05 | -0.11 | 0.00 |
| pNN50 (%) | 0.08 | -0.17 | 0.05 | -0.08 | -0.09 | -0.07 | -0.06 | -0.08 |
| LF (n.u.) | 0.04 | 0.14 | -0.18 | -0.12 | -0.12 | -0.04 | -0.13 | 0.10 |
| HF (n.u.) | -0.04 | -0.14 | 0.18 | 0.12 | 0.12 | 0.04 | 0.13 | -0.10 |
| Log LF/HF | 0.06 | 0.19 | -0.20 | -0.07 | -0.07 | -0.04 | -0.06 | 0.08 |
| SD1 (ms) | 0.05 | -0.20 | 0.09 | -0.06 | -0.07 | -0.04 | -0.06 | 0.01 |
| SD2 (ms) | 0.13 | -0.22 | -0.01 | -0.12 | -0.13 | -0.05 | -0.13 | 0.00 |
| SD2/SD1 | 0.05 | 0.12 | -0.27 | -0.04 | -0.04 | -0.07 | 0.00 | -0.04 |
| * p < 0.05 | | | | | | | | |
